# Preparation parameters of polyaniline/polyvinyl chloride flexible wires for electrical conductivity performance analysis based on orthogonal arrays

**DOI:** 10.1080/15685551.2021.1936373

**Published:** 2021-06-28

**Authors:** Wu Xuelian, Jiang Jiang, Yang Jian, Feng Qin, Wang Zhifeng

**Affiliations:** a School of Mechanical Engineering, Jiangsu University, Zhenjiang City, Jiangsu Province, China; b Mechanical and Electrical Engineering, Dazhou Vocational and Technical College, Dazhou City, Sichuan Province, China; c Testing Center, Yangzhou University, Yangzhou, PR China

**Keywords:** Polymer, polyaniline, flexible conductive wire, orthogonal test

## Abstract

A flexible polyaniline/polyvinyl chloride (PVC) polymer conductive wire was prepared using flexible PVC polymer as the substrate by the swelling – in-situ polymerization method, the line-shaped dents were pressed on the substrate by the thermodynamic pre-deformation treatment technology. Based on the orthogonal test method, the effects of five main influencing factors – swelling time (A), swelling temperature (B), oxidation temperature (C), oxidation time (D), and oxidant concentration (E) – on the conductivity of the prepared polyaniline/PVC conductive wire was investigated. The results of the orthogonal array testing were subjected to range analysis and analysis of variance (ANOVA), and the influencing factors, in terms of significance, follow the order of swelling temperature, oxidation time, swelling time, oxidation temperature, and oxidant concentration, with the optimal factor-level combination being A_2_B_2_C_2_D_2_E_2_, which led to a desirable conductivity up to 1.19 × 10^−1^ S/cm. In addition, the influence of different conductive line size characteristics on the molecular structure, microstructure, and conductivity of polyaniline/PVC flexible conductive wire was further studied. On the microstructure, as the line width increases, the infrared absorption intensity ratio of the quinone ring and the benzene ring in the polyaniline/PVC conductive wires gradually approaches 1. The microstructure, as the line width of the polyaniline/PVC conductive wire increases, the formed polyaniline gradually changes from flakes and granules to fibrous strips and entangles with each other to form a spatial network structure. The conductivity of the wire increases with the increase of its width up to 1.48 × 10^−1^ S/cm.

## 1. Introduction

Circuits formed by conductive wires made of organic or inorganic materials fabricated on a flexible or ductile substrate are called flexible loops, which form a flexible circuit when connected to the electronic components. Compared with traditional circuits, flexible circuits have better flexibility and deformability, can adapt to more complex working environments to a certain extent, and can meet the deformation requirements of wearable electronic devices and flexible electronics [1–4]. As an organic conductive polymer material, polyaniline is widely used in flexible electronics, sensors, energy storage, etc, due to its unique electrical properties, optical properties, stability, and biocompatibility [5–8]. The good electrical conductivity of polyaniline makes it have extremely bright application prospects in the field of electronic engineering.

The electrical conductivity of polyaniline is mainly determined by degree of oxidation and protonation doping. The oxidation state of polyaniline synthesized under different oxidation conditions and the conductivity of different oxidation states are very different; different oxidation degrees have a greater impact on the doping of protic acid; polyaniline in the intermediate oxidation state has a greater degree of conductivity after doping [9,10]. The molar ratio of the oxidation temperature, oxidation time, the concentration of the oxidizing agent, molar ratio of aniline and ammonium persulfate, the oxidant species, type of acid doping of the main factors of the polyaniline [11,12]. At present, most researches focus on the effect of single factor on the conductivity of polyaniline, and there are relatively few reports on the effect of multiple influencing factors on the conductivity of polyaniline at the same time. Orthogonal test is a method which studies multiple factors and levels. It selects representative level combinations from comprehensive experiments based on orthogonality for testing. These level combinations have the characteristics of uniform dispersion and strong comparability. After the indicators of performance test for data analysis and processing can be drawn from the main factors are optimal level combination, economically efficient, and fast characteristics [13–15].

In this study, flexible PVC polymer substrate, pre-deformation processing by pressing thermodynamic dent on the base line, by swelling – situ polymerization of polyaniline /PVC flexible conductive wire. Based on the orthogonal experiment method, the effects of five main influencing factors: swelling time (A), swelling temperature (B), oxidation temperature (C), oxidation time (D), and oxidant concentration (E), on the conductive properties of polyaniline/PVC conductive wire were investigated. Perform range analysis and variance analysis on the results of the orthogonal experiment to obtain the best electrical conductivity level combination; on the basis of this parameter, polyaniline/PVC conductive wire with different wire widths were further prepared to characterize the molecular structure, microscopic morphology, and conductivity of the conductive wire which analyze the influence of different widths on their molecular structure, micro morphology, and conductivity.

## 2. Tests and principles

### 2.1 Test materials and scheme

A 2 mm thick PVC flexible polymer was chosen for this study, from Guangdong Dongguan Tiansheng Plastic Co., Ltd., which has good electrical insulation and mechanical flexibility. The reagents used for aniline (analytical grade), ammonium persulfate (analytical grade), hydrochloric acid (analytical grade), and absolute ethanol (analytical grade) are all from Sinopharm Chemical Reagent Co., Ltd.

Polyaniline/PVC conductive wire preparation process: first, the substrate placed in a mold heated to 100°C (deformation temperature should be slightly higher than the glass transition temperature of PVC 90°C), then apply pressure to form a 2 mm × 1.5 mm × 30 mm wire groove on the substrate, keep the external force to cool to room temperature, take out the substrate, and then introduce the aniline monomer solution into the groove to swell the material in the groove area. After the adsorption time taken to reach the swelling solution of aniline remaining in the groove, then introducing ammonium persulfate oxidant electrode is oxidized, doped polyaniline line after oxidation molding, drying treatment performed after doping 30 °C. Subsequently, aniline monomer was introduced into the line groove for swelling and in-situ polymerization. After swelling for a specific duration, the surface layer of the unreacted aniline was absorbed, and the oxidant, an acidic solution of ammonium persulfate, was introduced for oxidation. After the completion of oxidation, the obtained polyaniline wire was doped and dried at 30 °C.

The experiments were conducted based on orthogonal arrays, with the conductivity of polyaniline/PVC flexible conductive wires as the assessment target. The effects of the five main influencing factors – swelling time, swelling temperature, oxidation time, oxidant concentration, and oxidation temperature – on the conductivity of the prepared flexible polyaniline/PVC conductive wires were investigated. A 5-factor-5-level orthogonal array test was designed, with design orthogonal parameters as shown in Table 1.

**Table 1.** 5-factor-5-level parameters

Level	A	B	C	D	E
1	1	0	0	10	0.5
2	3	10	10	30	1
3	6	20	20	60	1.5
4	12	30	30	120	2
5	15	40	40	180	2.5

A: swelling time (h), B: swelling temperature (°C), C: oxidation temperature (°C), D: oxidation time (min), E: oxidant concentration (mol/L).

The results of the orthogonal array test were analysed, and the level parameters were optimized. Based on these parameters, the molecular structure, microscopic morphology, and conductivity of the flexible conductive wires of polyaniline/PVC with different linewidths (0.5 mm, 1 mm, 1.5 mm, 2 mm, 2.5 mm) were further investigated. Moreover, the influences of the different linewidths on the molecular structure, microscopic morphology, and conductivity of the conductive wires were analysed.

### 2.2 Performance testing and characterization

The electrical conductivity of the conductive wires was tested using a digital 4-probe tester (ST2263, Suzhou Jingle Electronics Co., Ltd.). Attenuated total reflection Fourier transform infrared spectroscopy (ATR-FTIR, Nicolet AVATAR360, Madison) characterization of the structural properties of the molecule of polyaniline, grind polyaniline/PVC and potassium bromide and press them into tablets, and then perform infrared curve test. The morphology was characterized by a field emission scanning electron microscope (SEM, JSM-7800, Hitachi), and the surface of the material was sprayed with gold to observe the micro morphology of the conductive wire of polyaniline.

## 3. Results and discussion

### 3.1 Results of the orthogonal array testing

The 5-factor-5-level orthogonal arrays and the target conductivity are shown in Table 2, and range analysis and Analysis of variance (ANOVA) were conducted for the test results. ANOVA was carried out with a single variable using the general linear model of SPSS software to calculate and analyse whether the five factors significantly influence the electrical conductivity of the flexible polyaniline/PVC conductive wires.

**Table 2.** 5-factor-5-level orthogonal arrays and target results

Number	A	B	C	D	E	Conductivity S/cm
1	1	1	1	1	1	3.39 × 10^−2^
2	1	2	3	4	5	6.93 × 10^−2^
3	1	3	5	2	4	7.61 × 10^−2^
4	1	4	2	5	3	1.25 × 10^−2^
5	1	5	4	3	2	3.15 × 10^−3^
6	2	1	5	4	3	4.64 × 10^−2^
7	2	2	2	2	2	1.19 × 10^−1^
8	2	3	4	5	1	4.28 × 10^−2^
9	2	4	1	3	5	3.80 × 10^−4^
10	2	5	3	1	4	1.85 × 10^−3^
11	3	1	4	2	5	5.30 × 10^−2^
12	3	2	1	5	4	7.99 × 10^−2^
13	3	3	3	3	3	2.28 × 10^−2^
14	3	4	5	1	2	3.50 × 10^−2^
15	3	5	2	4	1	9.21 × 10^−4^
16	4	1	3	5	2	6.85 × 10^−3^
17	4	2	5	3	1	2.33 × 10^−2^
18	4	3	2	1	5	1.65 × 10^−2^
19	4	4	4	4	4	1.19 × 10^−2^
20	4	5	1	2	3	3.17 × 10^−3^
21	5	1	2	3	4	2.83 × 10^−2^
22	5	2	4	1	3	4.56 × 10^−3^
23	5	3	1	4	2	2.33 × 10^−2^
24	5	4	3	2	1	4.02 × 10^−3^
25	5	5	5	5	5	3.78 × 10^−3^

Range analysis, also known as intuitive analysis, reveals the significance of factors and the optimal combination that influences the test indicator through simple calculations and analysis. In the orthogonal arrays, each factor has five levels, and the test is repeated five times for each level. The sum of the results of the five trials at that level is denoted by k_i_, and the result of that level is the average value, ${\overset{ˉ}{k}}_{i}\;=\;k_{i}/5$. The range, R indicates the difference between the maximum value (${\overset{ˉ}{k}}_{{\;}_{imax}}$) and the minimum value (${\overset{ˉ}{k}}_{{\;}_{i\min}}$) of the different levels for the same factor. The greater the value of R, the greater the influence of the factor on the target results. From a high R value, the main influencing factor can be deduced. The smaller the value of R, the smaller the influence, with the corresponding factor likely being a secondary factor. The formula for calculating the value of R is as follows:

$$R=\;{\overset{ˉ}{k}}_{i\max}-{\overset{ˉ}{k}}_{i\min}$$

#### 3.1.1 Visual analysis

There were 25 sets of tests on the following orthogonal arrays, and each test was repeated thrice. The target results were taken to be the average of the three tests. First, the orthogonal arrays and the target results were directly observed to determine the factor-level combination for optimal conductivity. The optimal conductivity 1.19 × 10^−1^ S/cm was obtained with sample test #7, which is the only test that reached the 10^−1^ S/cm order of magnitude, with the factor-level combination of A_2_B_2_C_2_D_2_E_2_.

#### 3.1.2 Range analysis

Range analysis was conducted for the test results; the analysis results and the optimal level parameters are shown in Table 3. The range analysis shows that the range, R, affecting the conductivity of the polyaniline/PVC conductive wire has a maximum value of 4.08 × 10^−2^, indicating that the main influencing factor is the swelling temperature. The R values of oxidation time and swelling time are smaller than that of the swelling temperature and close to each other, indicating that their influence on electrical conductivity is not as extensive as that of the swelling temperature. The R values of oxidation temperature and oxidant concentration are even smaller, indicating relatively small influence on conductivity, i.e., the two factors are secondary factors. Through comparing the magnitude of the R values, it can be concluded that the factors affecting conductivity are in the following order of significance: B > D > A > C > E, i.e., swelling temperature, followed by oxidation time, swelling time, oxidation temperature, and oxidant concentration.

**Table 3.** Range analysis

Evaluation	A	B	C	D	E
k_1_	1.95 × 10^−1^	1.69 × 10^−1^	4.06 × 10^−2^	9.19 × 10^−2^	1.05 × 10^−1^
k_2_	2.10 × 10^−1^	2.17 × 10^−1^	1.77 × 10^−1^	2.55 × 10^−1^	1.68 × 10^−1^
k_3_	1.13 × 10^−1^	1.60 × 10^−1^	1.05 × 10^−1^	7.79 × 10^−2^	8.95 × 10^−2^
k_4_	6.17 × 10^−2^	6.22 × 10^−2^	1.16 × 10^−1^	1.31 × 10^−1^	1.19 × 10^−1^
k_5_	4.30 × 10^−2^	1.29 × 10^−2^	1.85 × 10^−1^	6.68 × 10^−2^	1.43 × 10^−1^
${\overset{ˉ}{k}}_{1}$	3.90 × 10^−2^	3.37 × 10^−2^	8.12 × 10^−3^	1.84 × 10^−2^	2.10 × 10^−2^
${\overset{ˉ}{k}}_{2}$	4.21 × 10^−2^	4.33 × 10^−2^	3.54 × 10^−2^	5.10 × 10^−2^	3.36 × 10^−2^
${\overset{ˉ}{k}}_{3}$	2.25 × 10^−2^	3.21 × 10^−2^	2.10 × 10^−2^	1.56 × 10^−2^	1.79 × 10^−2^
${\overset{ˉ}{k}}_{4}$	1.23 × 10^−2^	1.24 × 10^−2^	2.31 × 10^−2^	2.62 × 10^−2^	2.38 × 10^−2^
${\overset{ˉ}{k}}_{5}$	8.60 × 10^−3^	2.57 × 10^−3^	3.69 × 10^−2^	1.34 × 10^−2^	2.86 × 10^−2^
Range R	3.35 × 10^−2^	4.08 × 10^−2^	2.88 × 10^−2^	3.77 × 10^−2^	1.57 × 10^−2^
Order of importance	B > D > A > C > E				

The value of ${\overset{ˉ}{k}}_{i}$ reflects the magnitude of the influence on the test indicator attributed to the factor at different levels; the larger the value of the target (conductivity), the better the performance. Hence, for the same factor, the largest ${\overset{ˉ}{k}}_{i}$ value corresponds to the optimal level of that factor. The optimal factor-level combination was ascertained to be A_2_B_2_C_5_D_2_E_2_, based on the range analysis.

**Figure 1.**
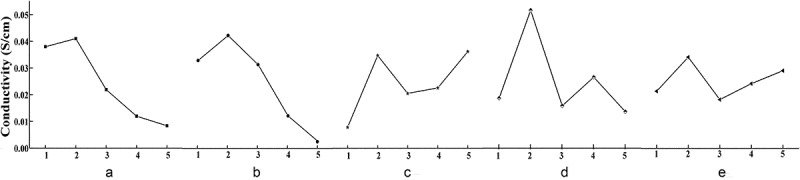
Trend of conductivity for different factor-level combinations

The patterns and trends of the effects of the different levels of the five factors (A, B, C, D, and E) on conductivity were analysed to optimize the factor-level combination. Figure 1 represents the trend of conductivity with the different levels of factors. With the change in level for factor A (swelling time), conductivity exhibits an increasing trend initially, followed by a decreasing trend, achieving the optimal conductivity at a swelling time of 3 h. The conductivity decreased after 3 h, i.e., the optimal level of swelling time was A_2_.

With the change in level for factor B (swelling temperature), the conductivity exhibited a similar trend of initial increase, followed by subsequent decrease, achieving an optimal value at a swelling temperature of 10 °C. When the swelling temperature increased above 10 °C, conductivity decreased at a rapid pace. Therefore, the optimal level of swelling temperature is B_2_.

With the change in level for factor C (oxidation temperature), the conductivity exhibited a trend resembling ripples. With an increase in the oxidation temperature, the conductivity first increases, then decreases, and increases again. The conductivity shows a surge when the oxidation temperature increases from 0 °C to 10 °C, while it increases gradually when the oxidation temperature increases from 20 °C to 40 °C. According to the results of the range analysis, it is clear that the oxidation temperature is a secondary influencing factor on the conductivity. Moreover, the values of ${\overset{ˉ}{k}}_{2}$ and ${\overset{ˉ}{k}}_{5}$ for oxidation temperature are essentially the same. Taking into consideration the ease of operation and manufacturing cost, the optimal level of oxidation temperature was chosen as C_2_.

With the change in level for factor D (oxidation time), the conductivity shows a hill-like, undulating trend. When the oxidation time is at level 2 (i.e., 30 min), the value of ${\overset{ˉ}{k}}_{2}$ is significantly greater than the ${\overset{ˉ}{k}}_{i}$ values at the other levels, indicating that the overall conductivity at level 2 is better than that at the other levels. Hence, the optimal level of oxidation time is D_2_.

With the change in level for factor E (oxidant concentration), the conductivity increases initially, then decreases, then increases again. Therefore, oxidant concentration is a minor influencing factor. The optimal level of oxidant concentration is E_2_, based on the relationship between the factor-level combination and conductivity and from the viewpoint of the manufacturing cost.

The factor-level combination for optimal conductivity is A_2_B_2_C_2_D_2_E_2_, based on the visual analysis method, while range analysis shows that the influencing factors of conductivity are in the order of B > D > A > C > E in terms of significance, and the optimal factor-level combination is A_2_B_2_C_5_D_2_E_2_. These two factor-level combinations were further tested and compared, and the results show that the conductivity obtained for A_2_B_2_C_2_D_2_E_2_ was higher than that of A_2_B_2_C_5_D_2_E_2_. In view of the significance of the influencing factors, the comparison of the conductivity attributed to the two optimal factor-level combinations, the manufacturing cost, and the ease of operation, the optimal factor-level combination was concluded to be A_2_B_2_C_2_D_2_E_2_.

#### 3.1.3 Analysis of variance

ANOVA was carried out using the general univariate linear model of SPSS software to calculate and analyse whether the five factors (swelling time, swelling temperature, oxidation time, oxidant concentration, and oxidation temperature) have significant effects on the conductivity of the flexible polyaniline/PVC conductive wires. The ANOVA results shown in Table 4 reveal that the five factors (A, B, C, D, and E) have no significant effects on the conductivity (with a sig. value greater than 0.05). The order of influencing factors in terms of significance was B > D > A > C > E, which is consistent with the conclusion of ANOVA.

**Table 4.** Analysis of variance table

Dependent variable: conductivity					
Source	Type III sum of squares	df	mean square	F	Sig.
Calibration model	^.^ 018a	20	.001	1.472	.386
Intercept	.016	1	.016	24.745	.008
Swelling time	.005	4	.001	1.840	.285
Swelling temperature	.006	4	.001	2.225	.229
Oxidation temperature	.003	4	.001	1.107	.462
Oxidation time	.005	4	.001	1.889	.276
Oxidant concentration	.001	4	.000	.297	.867
Error	.003	4	.001		
Aggregate	.036	25			
Total aggregate of calibration	.021	24			

^a^R-squared = .880 (adjusted R-squared = .282)

In view of the findings of the range analysis and ANOVA, the optimal factor-level combination for the conductivity performance of the polyaniline/PVC conductive wires was determined to be A_2_B_2_C_2_D_2_E_2_. The conditions of the optimal factor-level combination are as follows: aniline was allowed to swell in the area of the indented lines at 10 °C for 3 h, oxidized with 1 mol/L ammonium persulfate acidic solution for 30 min at an oxidation temperature of 10 °C until it imbibed the shape of the mould. Following this, it was doped with 0.5 mol/L hydrochloric acid for another 30 min, and finally dried in an oven at 25 °C for 24 h.

Preparation of optimal parameters of the conductivity obtained polyaniline/PVC conductive wires through the range and variance analysis. In order to expand the application range of its conductive wires, based on the conclusions of range and variance analysis, the influence of line width on the molecular structure, microscopic morphology, and conductivity of conductive wires was further studied.

### 3.2 Molecular structure analysis of polyaniline conductive wires

**Figure 2.**
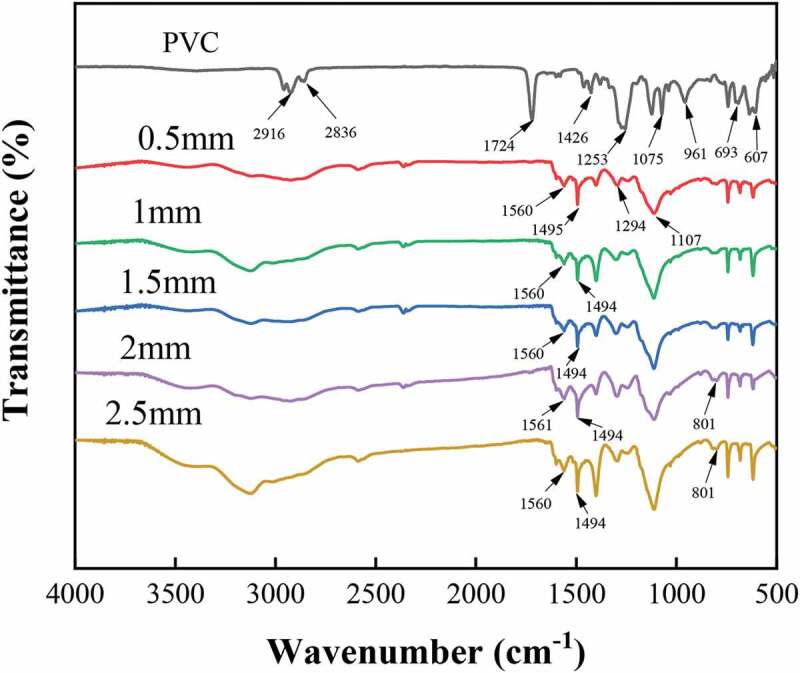
Infrared spectra of PVC and PANI/PVC conductive wire

Figure 2 shows the IR spectral profiles of PVC and the conductive wires of different linewidths. In the IR spectral profiles of PVC, the asymmetric stretching vibration peak of -CH- occurs at 2916 cm^−1^, the symmetric stretching vibration peak of -CH- occurs at 2836 cm^−1^, and the stretching vibration peak of C = O occurs at 1724 cm^−1^ due to the addition of the impact modifier acrylate (CH2 = CHCOOR) in PVC. Furthermore, the bending vibration peak of CH_2_ occurs at 1426 cm^−1^, the symmetric stretching vibration peak of C-H occurs at 1253 cm^−1^, the stretching vibration peaks of C-C occur at both 1124 cm^−1^ and 1075 cm^−1^, the trans-rocking characteristic peak of C-H occurs at 961 cm^−1^, and the stretching vibration peaks of C-Cl occur at both 693 cm^−1^ and 607 cm^−1^. The above-mentioned characteristic peaks correspond to the main molecular structure composition in the PVC molecular structure, that is, the infrared characteristic absorption of chemical bonds such as C-H, CH_2_, C-C, and C-Cl.

It is evident from the IR spectral profiles of the conductive wires with different linewidths that the IR spectral profiles of polyaniline with different linewidths are essentially the same. The quinone ring backbone vibration peak occurs at 1560 cm^−1^, the benzene ring backbone vibration peak occurs at 1494 cm^−1^, the N-B-N stretching vibration peak occurs at 1294 cm^−1^, the benzene ring in-plane bending vibration peak occurs at 1107 cm^−1^, and the 1,4-substituted benzene ring C-H out-of-plane bending vibration peak occurs at 801 cm^−1^, indicating that the polyaniline is polymerized in the form of head-to-tail bonding, this structure is beneficial to improve the electrical conductivity of the wire. Meanwhile, with the increase in linewidth, the absorption intensity of the characteristic peaks of quinone ring and benzene ring changed. This can be used to analyse the oxidation state of polyaniline qualitatively, and the specific ratios are shown in Table 5. With the increase in linewidth, the ratio of absorption peak intensity between the quinone ring (*I_q_*) and the benzene ring (*I_b_*) gradually decreased and stabilized at 1. This is when the polyaniline in the intermediate oxidation state was formed, and the conductivity can increase greatly after doping. Because the conductive substance of polyaniline is formed via in-situ polymerization through the swelling-oxidation on the PVC substrate, the infrared characteristic peak of polyvinyl chloride in the polyaniline/PVC conductive wire is weakened and no new infrared characteristic peaks appeared. This indicates that the surface polyaniline did not react with PVC. Since the conductive polyaniline was swelled and oxidized in-situ polymerization in the dent area of the polyvinyl chloride body, and the density of the dent surface is relatively high, the infrared characteristic peak of the polyvinyl chloride in the polyaniline/polyvinyl chloride conductive line is weakened.

**Table 5.** Intensity ratios of the quinone and benzene rings with different line-width

Line-width	*I_q_*	*I_b_*	*I_q_*/*I_b_*
0.5	3353.6	2718.4	1.23
1	3288.5	2751.8	1.19
1.5	2914.9	2855.9	1.02
2	3071.6	3298.9	0.93
2.5	3192.8	3070.3	1.04

### 3.3 Microscopic morphology of polyaniline conductive wires

**Figure 3.**
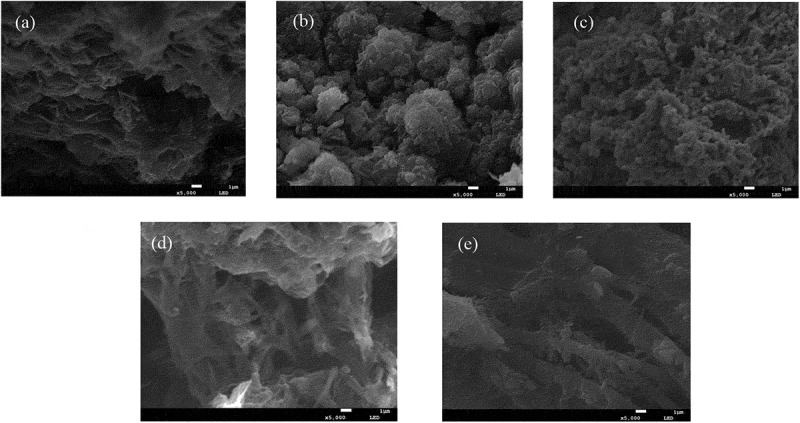
SEM morphology of polyaniline conductive wires with different linewidths (a: 0.5 mm, b: 1 mm, c: 1.5 mm, d: 2 mm, e: 2.5 mm)

Figure 3 shows the microscopic morphology of polyaniline/PVC conductive wires. Figure 3(a) shows the microscopic morphology of polyaniline with 0.5 mm linewidth, observed in the form of agglomerates in a lamellar structure with numerous short, rod-like substances, presumed to be short and thin polyaniline oligomers. It can be seen from Figure 3(b) that the polyaniline in the polyaniline/PVC conductive wire with a line width of 1 mm is in the form of clusters of large particles, and the presence of rod-shaped aniline oligomers can still be observed. The polyaniline in the 1.5 mm line width polyaniline/PVC conductive circuit shown in Figure 3(c) shows a fine and dense granular morphology, and at the same time, the particle surface has a partial long dendritic morphology, but there is no interconnection. It is speculated that this is also an oligomer that forms polyaniline. Figure 3 (d) 2 mm line width shown polyaniline/PVC morphology of conductive wires are much more elongated and entangled, but there are also discontinuous polyaniline chain molecules. The polyaniline/PVC conductive wires with a line width of 2.5 mm shown in Figure 3(e) has a continuous strip structure and is closely intertwine. However, there are fewer granular or short rod-shaped aniline oligomers on the polyaniline macromolecular chain. In summary, as the line width of the polyaniline/PVC conductive wires increases, the formed polyaniline gradually changes from flakes and granules to fibrous strips and entangles with each other to form a spatial network structure. This is conducive to the construction of a stable conductive pathway.

### 3.4 Conductivity of polyaniline conductive wires

**Figure 4.**
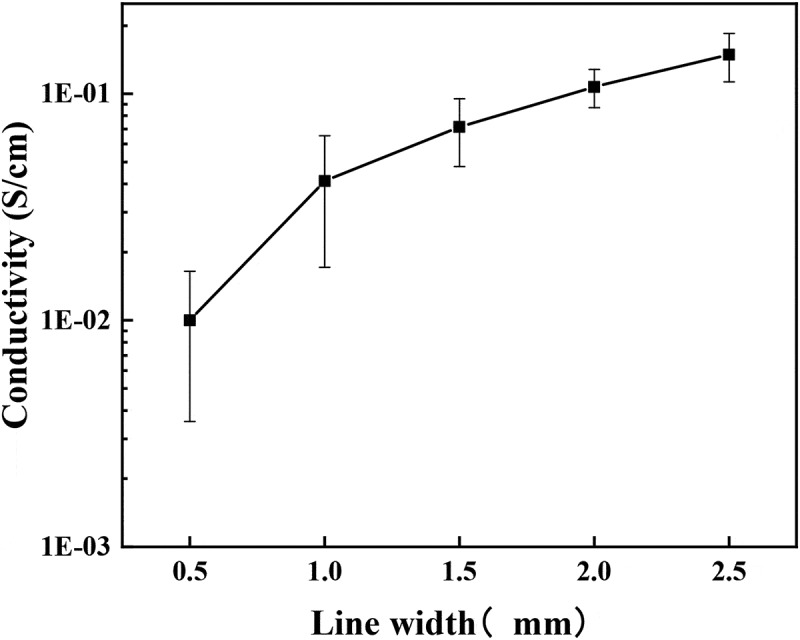
Diagram of different line-width and electrical conductivity

Figure 4 is the relationship between linewidth and conductivity, it is evident that the conductivity of the polyaniline conductive wires gradually increases with increasing linewidth; particularly, the conductivity increases the most when the linewidth increases from 0.5 mm to 1 mm. From the morphology and molecular structure, it can be observed that a lamellar structure with polyaniline oligomers is formed on the surface layer of the prepared polyaniline wire with 0.5 mm linewidth. Thus, it can be deduced that the presence of certain structural defects hinders the formation of a continuous and stable conductive pathway to allow the transition of carriers between molecular chains. For polyaniline wires with 1 mm and 1.5 mm linewidths, the surface is granular, the structure of the molecular chains is relatively dense, and the conductivity is improved somewhat. However, the conductive model between the particles resembles multiple metallic islands where the conductive pathway is discontinuous and unstable [16,17]. When the linewidth reaches 2 mm, the conductivity of the conductive wire increases by an order of magnitude to 10^−1^ S/cm, possibly due to the fibrous structure of the polyaniline formed based on the microstructure, which is conducive to the construction of stable conductive pathways between the polyaniline molecular chains. The microstructure with 2.5 mm linewidth is conducive to the effective doping of hydrochloric acid, further improving the electrical conductivity of the polyaniline wires.

## 4. Conclusion

Flexible polymeric substrate is PVC, pre-deformation processing by pressing thermodynamic dent on the base line, by swelling – situ Polymerization of polyaniline/PVC flexible conductive wire. The results of the orthogonal array testing were subjected to range analysis and analysis of variance (ANOVA), and the influencing factors, in terms of significance, follow the order of swelling temperature, oxidation time, swelling time, oxidation temperature, and oxidant concentration, with the optimal factor-level combination being A_2_B_2_C_2_D_2_E_2_. Based on these parameters, the polyaniline conductive wires with different linewidths were prepared, and their molecular structures, microscopic morphology, and conductivity were analysed to study the influence of linewidth on the conductivity of the conductive wires. The results show that the oxidation states of polyaniline with different linewidths are somewhat different. A sheet-like morphology of polyaniline, the particulate gradually transformed into fibers and intertwining stripe formed network structure, formed polyaniline chain molecules facilitates to build a stable conductive path. In particular, the polyanilines with 2 mm and 2.5 mm linewidths are in the intermediate oxidation state, with the microscopic morphology resembling long and tangled stripes, and the conductivity can reach up to 10^−1^ S/cm after doping.
